# Sir Joseph Lister on Antisepsis

**Published:** 1893-07

**Authors:** 


### SIR JOSEPH LISTER ON ANTISEPSIS.

In a late lecture Mr. Lister announced his views and described his present practice with respect to surgical antisepsis. In it we witness a departure from troublesome and complicated methods and a return to the simpler.

In the first place, he has now practically abandoned the once necessary bichloride of mercury. It has been found by experiment that the tubercle bacillus is much less capable of resisting a carbolic than a bichloride solution. The sponges, the instruments and the field of operation are cleansed and sterilized in a carbolic solution, generally 5 per cent. For absorbents, Mr. Lister still employs sponges, though the latter are regarded by many as the frequent carriers of septic organisms. Sterilized tissues, such as gauze and cotton, have less absorptive capacity, and cannot take the place of sponge. His sponges are first washed with soap and water; then with soda; then washed again and dried; and then steeped in a 5 per cent. carbolic solution, when they are ready for use. After an operation the sponges are placed in a tank of water, and the fibrin clinging to them allowed to putrefy. They are then thoroughly washed and put away in the carbolic solution.

Mr. Lister thinks it entirely unnecessary to keep the skin soaked in an antiseptic lotion for several hours previous to an operation. To cleanse the parts he used simply the 5 per cent. carbolic solution; he does not even use soap and water. Carbolic acid has a greater affinity for the epidermis than corrosive sublimate, combining in all proportions with the fatty matters of the same.

The elaborate methods of sterilizing instruments by boiling are troublesome and unnecessary. In his own practice he begins to place his instruments into the carbolic solution just as the patient is brought into the room. By the time all the preparations (including anæsthesia) are complete, the sterilization is accomplished.

Iodoform continues to be the best antiseptic application, though it seems to have little effect upon the development of bacteria. Its potency seems to be due to its chemical action upon the products of the bacteria. Iodoform is specially valuable in the interior of wounds. An external dressing of iodoform gauze, when it becomes soiled with wound discharges, will not prevent the development of bacteria.

The perfect external antiseptic dressing should have these four qualities:

1. It should contain some thoroughly reliable antiseptic ingredient.

2. That substance should be of such a character as not to be dissipated to a dangerous degree before the dressing is changed.

3. It should be unirritating.

4. It should be a good absorbent of discharges.

Sir Joseph has found the double cyanide of mercury and zinc to meet these indications better than any other agent.

It is a significant point that this great apostle of antisepsis is returning from the complicated and cumbersome methods to the more simple. The results which he now gets are said not to be surpassed by those who observe antiseptic precautions the most rigid.

---

THE Atlanta Obstetrical Society has been organized with a membership of earnest and enthusiastic workers. Drs. V. O. Hardon, Geo. H. Noble, W. A. Crow, R. R. Kime, J. C. Avary and E. C. Davis compose the present membership, but others will be added from time to time who are especially interested in obstetrical and gynecological work. The next meeting of the society will be held July 13th, when Dr. Noble will read a paper on the treatment of abortion. Members of the profession who desire to do so are cordially invited to attend the meetings. The papers and discussions of this society will be full of interest for the general profession, and we will take pleasure in reporting them for our readers.

---

WE regret to learn of the death of Dr. R. O. Engram, of Montezuma. Dr. Engram was an excellent and beloved physician, and no one stood higher in the profession. At the time of his death he was editor of the Montezuma *Record*.

